# IgG4-related sclerosing mesenteritis causing bowel obstruction: a case report

**DOI:** 10.1186/s40792-016-0248-0

**Published:** 2016-10-30

**Authors:** Atsushi Abe, Tatsuya Manabe, Nobuyoshi Takizawa, Takashi Ueki, Daisuke Yamada, Kinuko Nagayoshi, Yoshihiko Sadakari, Hayato Fujita, Shuntaro Nagai, Hidetaka Yamamoto, Yoshinao Oda, Masafumi Nakamura

**Affiliations:** 1 Department of Surgery and Oncology, Graduate School of Medical Sciences, Kyushu University, Maidashi 3-1-1, Higashi-ku, Fukuoka, 812-8582 Japan; 2 Department of Anatomic Pathology, Graduate School of Medical Sciences, Kyushu University, Fukuoka, 812-8582 Japan; 3 Department of Surgery, Hamanomachi Hospital, Fukuoka, 810-8539 Japan; 4 Department of Surgery, Japan Community Health Care Organization, Kyushu Hospital, Fukuoka, 806-8501 Japan

**Keywords:** IgG4-related SM, Sclerosing mesenteritis, Intestinal obstruction

## Abstract

Sclerosing mesenteritis (SM) is a rare inflammatory and fibrosing disease primarily involving the small-bowel mesentery. Recently, SM was reported to be closely related to IgG4-related disease (IgG4-RD). This report describes a patient with SM associated with IgG4-RD. A 77-year-old woman with a history of surgery for ectopic pregnancy and wound dehiscence presented with intestinal obstruction. Abdominal enhanced computed tomography (CT) revealed an enhanced, radially shaped, oval mass, 3 cm in diameter, with an unclear rim in the mesentery of the distal ileum, which may have involved the distal ileum. To remove the cause of bowel obstruction, the SM was resected completely and the ileum was resected partially. Histologic examination showed that the mass was composed of spindle cells arranged in a fascicular or storiform pattern; moreover, fibrous stroma was observed, with dense lymphoplasmacytic infiltration and lymphoid follicles. Immunohistochemically, numerous IgG4-positive plasma cells were observed, at a density of 253 per high-powered field, and the IgG4/IgG ratio was about 50 %. Elastica van Gieson (EVG) staining also showed obstructive phlebitis. These findings indicated IgG4-related SM. Although the accurate diagnosis of SM remains difficult without histological analysis, IgG4-RD should be included in the differential diagnosis of unknown mesenteric tumors. Identification of IgG4-RD may prevent unnecessary surgery because corticosteroids may be effective in these patients.

## Background

Sclerosing mesenteritis (SM) is a rare inflammatory and fibrosing disease of unknown etiology that primarily involves the small-bowel mesentery, most frequently observed in middle-aged and older men [1–6]. SM, also called mesenteric fibrosis, mesenteric lipodystrophy, and retractile mesenteritis, is histologically characterized by varying degrees of fibrosis, chronic inflammation, and fat necrosis [1]. On imaging, SM appears as a well- or ill-defined mass in the mesentery, which may be clinically misdiagnosed as a malignant neoplasm [7–9].

SM was recently reported to be closely related to IgG4-related disease (IgG4-RD) [2, 10–13], a systemic syndrome characterized by masses in various organs infiltrated by IgG4-positive plasma cells and high serum IgG4 concentrations [14, 15]. This report describes a patient with IgG4-related SM causing bowel obstruction.

## Case presentation

A 77-year-old woman, who had a history of surgery for ectopic pregnancy and wound dehiscence 28 years earlier, presented to another hospital with intermittent abdominal pain. She was diagnosed with an intestinal obstruction and admitted to the hospital. Computed tomography (CT) revealed an irregularly shaped mass, 3 cm in diameter, in the mesentery of the ileum, which was suspected of causing her bowel obstruction. Medical treatment, including long-tube decompression, improved her symptoms, and she was referred to our hospital for further examination and treatment.

**Fig. 1:**
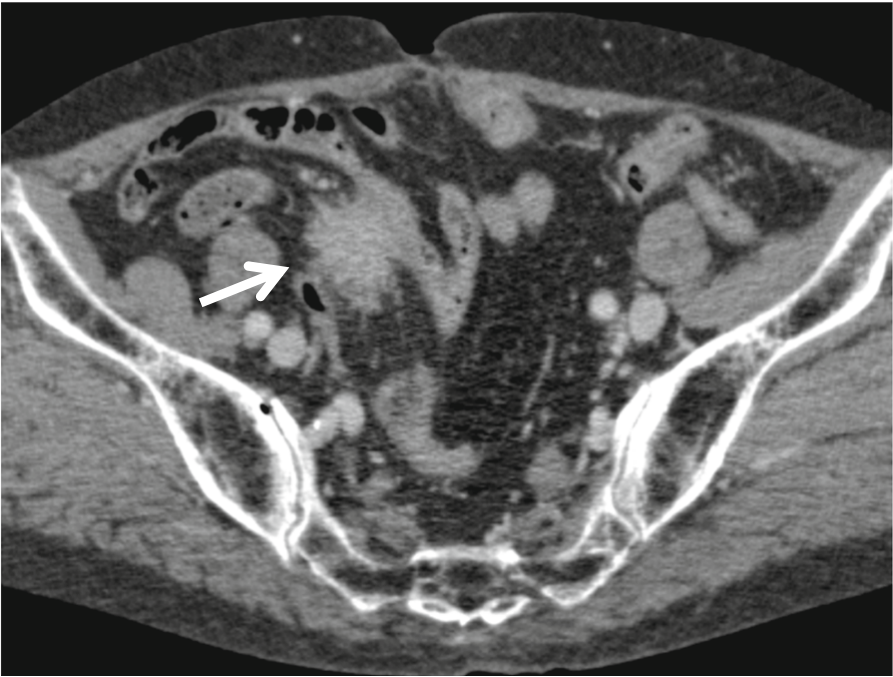
Abdominal enhanced CT, showing a radial, irregularly shaped mass (*white arrow*), 26 mm in diameter, at the root of the mesentery in the right lower quadrant and located close to the ileum

**Fig. 2:**
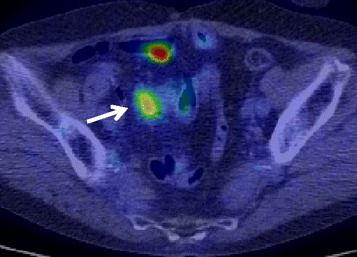
PET-CT scan, showing abnormal uptake of fluorodeoxyglucose by the mass (*white arrow*; SUVmax = 4.4)

On admission to our hospital, she had no symptoms and a physical examination showed no abnormalities. Laboratory tests, including those for tumor markers such as CEA and CA19-9, showed no abnormalities. Her serum IgG4 concentration was not measured. Abdominal enhanced CT imaging revealed an enhanced, radially shaped, oval mass, 3 cm in diameter, with an unclear rim in the mesentery of the distal ileum, which may have involved the distal ileum (Fig. 1). Double balloon enteroscopy and gastrografin enterography showed no abnormal findings. As seen in Fig. 2, 18F-fluorodeoxyglucose positron emission tomography (FDG-PET) revealed a slight uptake of fluorodeoxyglucose (standardized uptake value 4.4) by the mass. These findings could not yield an accurate diagnosis, including whether the mass was malignant or inflammatory.

**Fig. 3:**
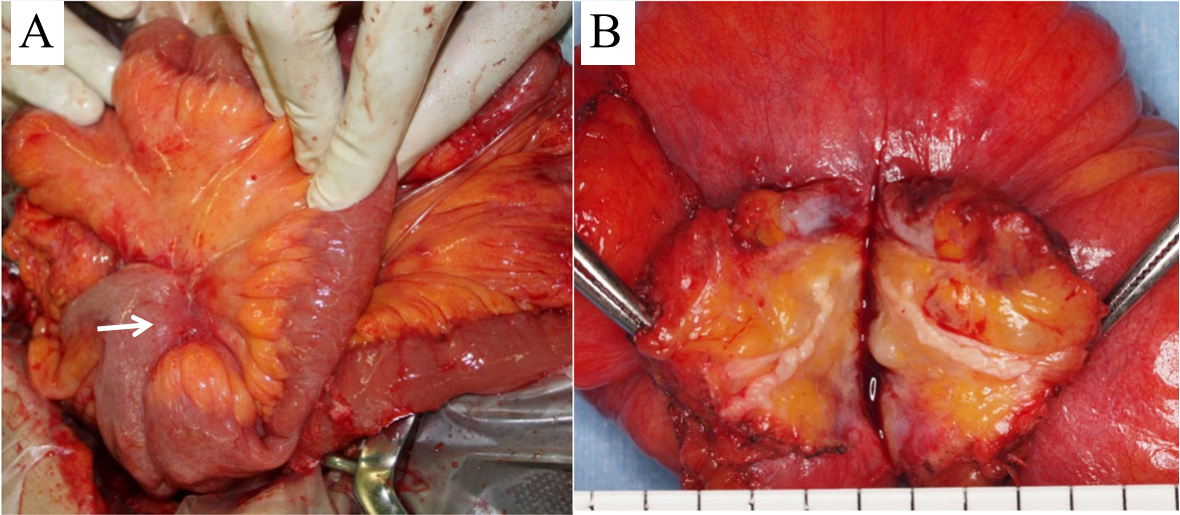
Intraoperative findings in the patient. **a** An elastic soft, yellowish white-like mass (*arrow*) was observed at the root of the mesentery, with the mass also involving the adjacent ileum. **b** View of the cut surface of the resected specimen, showing inflammatory fat tissue containing white fibrous strands

It was therefore decided to perform surgery, both to remove the cause of bowel obstruction and diagnose it pathologically. A midline incision was made, along with careful adhesiolysis for the tight adhesions over almost the entire intra-abdominal space resulting from the previous surgery. An elastic-hard yellowish mesenteric mass, involving the adjacent ileum, was detected (Fig. 3a). A partial ileal resection that included the mass was performed, followed by hand-sewn ileo-ileal anastomosis.

**Fig. 4:**
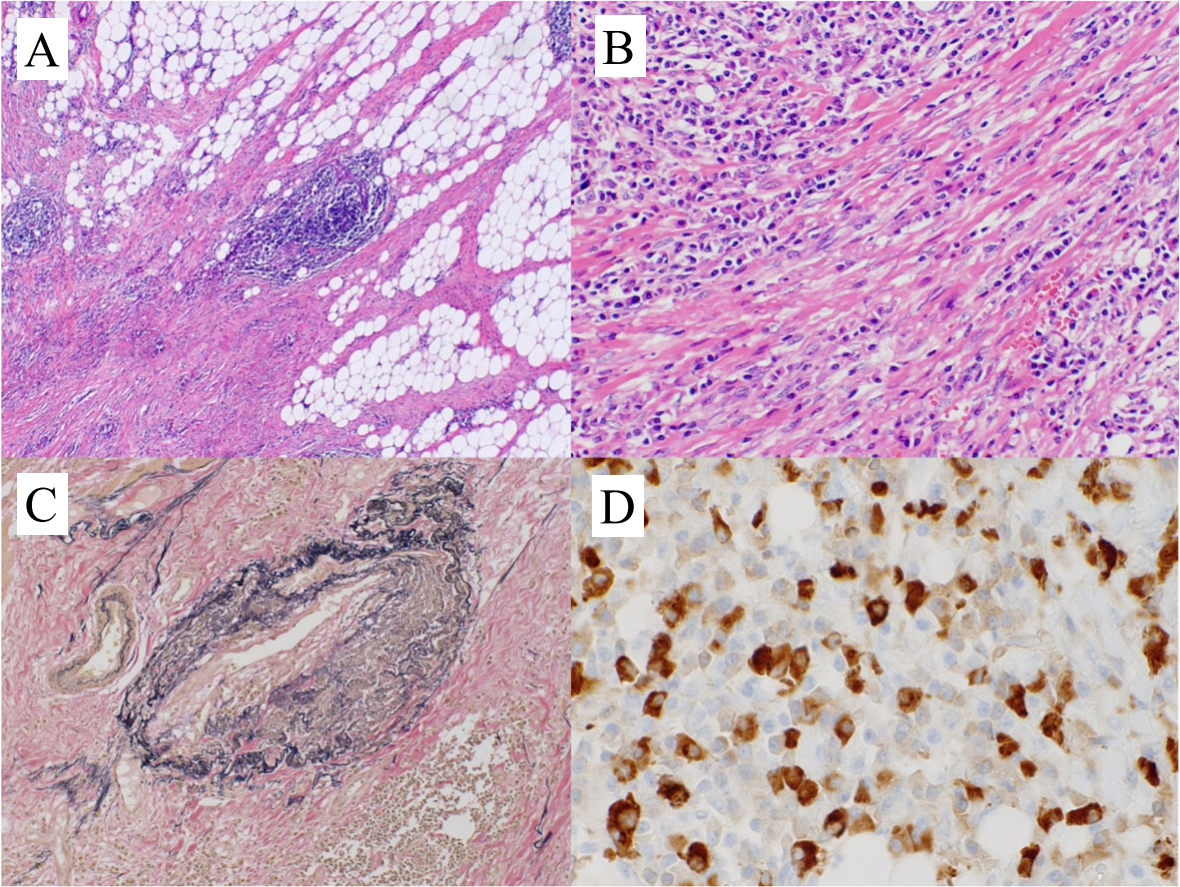
Histological examination of tissue specimens. **a**, **b** Hematoxylin and eosin staining, showing that the mass was composed of **a** prominent lymphoid follicles with sclerosis and **b** fascicular or storiform proliferation of spindle cells with inflammatory cells. **c** Elastica van Gieson (EVG) staining, showing obliterative phlebitis. **d** Immunohistochemical staining with anti-IgG4 antibody, showing that numerous IgG4-positive plasma cells were observed (253 per high-powered field (HPF); IgG4/IgG ratio = 50 %)

Macroscopic examination showed a radially shaped tumor with an unclear rim at the root of the mesentery. The cut surface of the resected specimen showed inflammatory fat tissue containing white fibrous strands (Fig. 3b). Microscopically, the mass was composed of spindle cells in a fascicular or storiform pattern and fibrous stroma with lymphoplasmacytic infiltration and lymphoid follicles (Fig. 4a,b). Elastica van Gieson (EVG) staining showed obstructive phlebitis (Fig. 4c). Immunohistochemical examination showed numerous IgG4-positive plasma cells, with an IgG4/IgG ratio of 50 % (Fig. 4d). Immunohistochemically, the spindle cells were negative for anaplastic lymphoma kinase (ALK), desmin, CDK4, MDM2, CD21, CD35, and nuclear β-catenin, whereas the lymphocytes in inter-lymphoid follicles were positive for CD3 and the lymphocytes in germinal centers were positive for CD20. These findings indicated a diagnosis of IgG4-related SM, while excluding diagnoses of leiomyosarcoma, inflammatory myofibroblastic tumor, desmoids tumor, liposarcoma, and follicular dendritic tumor. The patient’s serum IgG4 concentration 19 days after surgery was 114 mg/dl (normal range, 4.8–105 mg/dl). Further postoperative examination showed no evidence of IgG4-RD of other organs, including the pancreas and salivary glands. After follow-up for 4 years, there has been no evidence of SM recurrence or symptom relapse.

### Discussion

This report describes a patient with symptomatic SM as a manifestation of IgG4-RD. Because of its rarity, the etiology of SM remains undetermined [1–6]. Suggested causes include trauma (including surgery), powder on surgical gloves, infection (such as tuberculosis), autoimmune diseases, vascular insufficiency, and retained suture material [1–3]. About 40 to 70 % of patients with SM were found to have undergone previous surgery [1, 2]. Similarly, the patient described in this report had a history of surgery for ectopic pregnancy and wound dehiscence 28 years earlier. During surgery for SM, tight adhesions were seen throughout her abdomen. The pathogenic mechanism of SM seems to be a non-specific response to a wide variety of stimuli.

SM may be an IgG4-RD [10–13], diseases that dramatically respond to corticosteroid treatment [14, 15]. Recently, IgG4-RD was reported to be closely related to multifocal fibrosclerosis [14]. IgG4-RD is characterized by organ enlargement and nodular/hyperplastic lesions in various organs, either concurrently or metachronously, due to marked infiltration of lymphocytes and IgG4-positive plasma cells, as well as to fibrosis of unknown etiology [14, 15]. Although the incidence of SM related to IgG4-RD has not been determined, SM was observed in 2 (4 %) of 57 patients with autoimmune pancreatitis [3] and marked infiltration of IgG4-positive plasma cells was observed in 4 (33 %) of 12 patients with SM [2]. The comprehensive diagnostic criteria for IgG4-RD [15] require imaging and serum and histopathological examination. (1) Clinical examination should show characteristic diffuse/localized swelling or masses in single or multiple organs. (2) Hematological examination should show elevated serum IgG4 concentration (*≥*135 mg/dl). (3) Histopathologic examination should show marked lymphoplasmacytic infiltration, lymphoid follicles, obstructive phlebitis, dense fibrosis, and infiltration of IgG4-positive plasma cells.

The rate of IgG4- and IgG-positive cells diagnostic of IgG4-RD has been defined as *>*40 to 50 %, with >60 to 100 IgG4-positive cells present per high-powered field (HPF) [16]. The degree of infiltration of IgG4- and IgG-positive plasma cells is analyzed in areas with the highest density of positive cells, with three HPFs evaluated in each patient and averaged [16]. The postoperative serum IgG4 concentration in our patient was 114 mg/dl, higher than the normal range (4.8–105 mg/dl), but lower than the cutoff of 135 mg/dl. Thus, according to the above criteria, this patient should be diagnosed as having “probable” IgG4-RD.

A search of PubMed using “IgG4” and “sclerosing mesenteritis” as key words resulted in seven cases that seemed to be IgG4-related sclerosing mesenteritis [10–13, 17–19]. These seven cases and the present case are summarized in Table 1. The chief complaint was abdominal pain in five patients. Serum IgG4 levels were elevated in only three of eight patients, and the levels were not markedly elevated. Seven cases underwent resection because preoperative diagnosis was difficult. In almost all cases, abundant infiltration of IgG4-positive plasma cells and an elevated ratio of IgG4- and IgG-positive plasma cells (40%) was detected. Case 2, as diagnosed by biopsy, was successfully treated with steroids. Other organ involvement was not seen except for case 7.

**Table 1:** Summary of the clinicopathological features of IgG4-related sclerosing mesenteritis

Case	Age	Sex	Chief complaint	Sample	Size (cm)	Serum IgG4 (mg/dl)	Storiform fibrosis	Obliterative phlebitis	IgG4+ plasma cells count (/HPF)	IgG4+/IgG ratio (%)	Other IgG4-related disease	Steroid therapy (before resection)
1 [10]	46	M	NA	Resection	7	NA	−	NA	>100	<1/3	None	ND
2 [12]	42	M	Incidental	Resection	4	119	−	NA	60	40	None	ND
3 [11]	82	F	Abdominal pain	Resection	11.7	171^a^	−	+	130	75.9	None	ND
4 [13]	53	M	Abdominal pain	Resection	7	127^a^	+	+	74.8	64	None	ND
5 [17]	7	F	Abdominal pain	Biopsy	NA	149	−	NA	NA	52	None	Effective
6 [18]	64	M	Abdominal pain	Resection	6	81^a^	+	+	38	80	Retroperitoneal fibrosis	ND
7 [19]	70	F	Abdominal mass	Resection	7.9	213^a^	+	+	NA	>90	None	ND
Our case	77	F	Abdominal pain	Resection	2.6	114^a^	+	+	253	50	None	ND

*NA* not available, *ND* not done, *HPF* high-powered field

^a^The data after surgery

Most patients with SM are symptomatic, with abdominal pain or a palpable abdominal mass being the most common clinical manifestations [1, 2, 8]. SM symptoms are caused by a direct mechanical effect of the mass on the bowels, vessels, and lymphatics, resulting in abdominal pain, bowel obstruction, ischemia, and ascites. Abdominal CT scanning is important for an accurate diagnosis. In the absence of histological analysis, SM can be diagnosed by CT findings of (1) hyperattenuating mesenteric fat, especially at the root of the small-bowel mesentery, (2) well-defined soft tissue nodules less than 5 mm in diameter surrounded by a fatty halo (fat ring sign), and (3) a tumoral pseudo-capsule [7]. However, the imaging appearances of SM vary depending on the predominant tissue component (fat necrosis, inflammation, or fibrosis) [20]. Therefore, SM may still be very difficult to distinguish accurately from other mesenteric diseases, such as gastrointestinal stromal tumor, malignant lymphoma, metastatic carcinoid tumor, desmoid tumor, and metastatic adenocarcinoma [5, 7–9].

The histological differential diagnosis in our patient included leiomyosarcoma (desmin+), inflammatory myofibroblastic tumor (ALK+/−, IgG4−), desmoid fibromatosis (β-catenin nuclear+), liposarcoma (CDK4+, MDM2+), follicular dendritic tumor (CD21+, CD35+), and malignant lymphoma (T/B cell marker, light chain restriction). A definitive diagnosis of SM requires histological examination of biopsy or surgically excised tissue specimens, unless other organs are apparently affected by IgG4-RD or percutaneous needle biopsy can be easily performed.

No consensus has yet been reached for the treatment for SM. Asymptomatic or mildly symptomatic SM may be left untreated [1, 2]. Surgical exploration is advocated in patients with life-threatening complications, such as bowel obstruction or perforation, or if there is high suspicion of an alternative diagnosis, such as malignancy. Surgical intervention, predominantly incomplete resection of SM, did not resolve symptoms or prevent disease progression [2]. The effects of complete resection remain unknown, as complete resection is frequently prevented by vessel involvement. The patient described in this report underwent complete resection of SM and partial ileal resection, both for accurate diagnosis and for removal of the cause of bowel obstruction. Surgery resulted in good postoperative outcomes, without recurrence of the disease or symptoms after about 4 years of follow-up. Medical treatment, including with corticosteroids, tamoxifen, cyclophosphamide, and azathioprine, has also shown good results [1–3], although medical treatment for SM has not been standardized. If SM is a manifestation of IgG4-RD, corticosteroids may be promising.

## Conclusions

SM may be associated with IgG4-RD in some patients. Although the accurate diagnosis of SM remains difficult in the absence of histological examination, IgG4-RD should be included in the differential diagnosis of unknown mesenteric tumors. Corticosteroids may be effective in these patients, thereby avoiding unnecessary surgery.
